# Association between TGF-β1 and β-catenin expression in the vaginal wall of patients with pelvic organ prolapse

**DOI:** 10.1515/biol-2022-1058

**Published:** 2025-03-21

**Authors:** Feng-Qin Xue, Dan Xu, Shu-Rui Zhao, Ying Ma, Ye Zhao

**Affiliations:** The First Clinical Medical School, Shanxi Medical University, Taiyuan, 030001, China; Department of Gynecology, First Hospital of Shanxi Medical University, 85 South Jiefang Road, Taiyuan, 030001, Shanxi, China

**Keywords:** anterior vaginal wall, β-catenin, collagen, pelvic organ prolapse, TGF-β1

## Abstract

The aim of this study is to investigate the mechanism of action and correlation between transforming growth factor beta 1 (TGF-β1) and β-catenin in pelvic organ prolapse (POP). The study compared vaginal wall tissues from two groups: 20 patients with POP (POP group) and 20 who had hysterectomies for benign conditions (control group). Hematoxylin and Eosin and Masson staining visualized collagen, while TUNEL staining detected apoptosis. Protein and mRNA expression levels of TGF-β1, β-catenin, matrix metallopeptidase 2 (MMP2), tissue inhibitor of metalloproteinases 2 (TIMP2), and collagen, type I, alpha 1 (COL1A1) were assessed using immunohistochemistry, quantitative real-time polymerase chain reaction, and western blot techniques. Relationships between the protein expressions of TGF-β1 and β-catenin, β-catenin and COL1A1, and TGF-β1 and COL1A1 were analyzed. In the POP group, vaginal wall collagen fibers were sparse, disorganized, and fragmented, with fewer fibers and more apoptotic cells compared to the control group. Protein and mRNA levels of TGF-β1, β-catenin, TIMP2, and COL1A1 were significantly lower, while MMP2 was higher (*p* < 0.05). Positive correlations were found between TGF-β1, β-catenin, and COL1A1. Reduced TGF-β1 and β-catenin levels may trigger POP by affecting pelvic floor collagen metabolism.

## Graphical abstract

**Figure:**
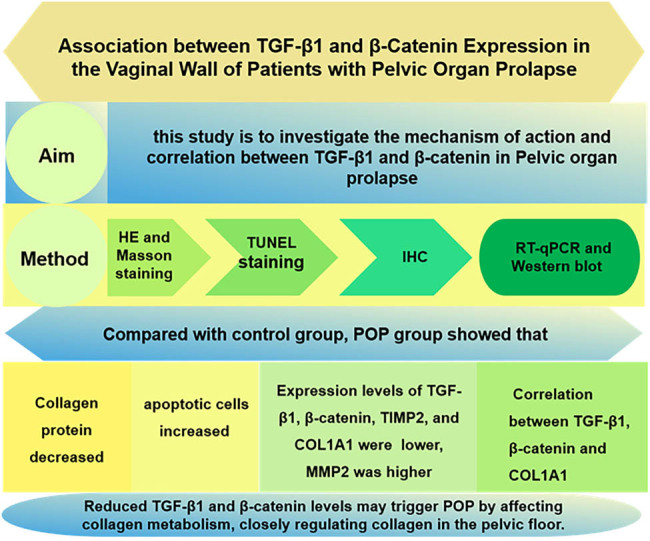

## 1 Introduction

Pelvic organ prolapse (POP) is a benign disorder caused by weakened pelvic floor support, characterized by the prolapse of the vaginal wall and/or uterus, often accompanied by fecal incontinence and dyspareunia [1]. Major risk factors for POP include vaginal delivery, aging, and menopause [2]. Although it rarely leads to serious morbidity and mortality, it has a significant impact on the physical and mental health of the patient. POP is most prevalent among individuals aged 60–69 [3]. Given the current national context in China, the incidence of POP is expected to rise, potentially exerting significant economic strain on the society. However, the underlying pathophysiology of POP has not been fully elucidated, and clinical management primarily focuses on symptomatic relief rather than addressing the root cause [4]. Therefore, we aim to investigate the molecular pathogenesis of POP to provide innovative insights for its accurate diagnosis and treatment.

The stability of pelvic organs relies on fibrous connective tissues. Consequently, any disruption or dysfunction within this connective tissue network can weaken the biomechanical properties of the pelvic floor support structures, potentially contributing to the development of POP.

Collagen is the primary structural component of the connective tissue [5], constituting 70–80% of connective tissue, and is the main element of the extracellular matrix. Collagen, which provides both strength and elasticity to tissues, serves as the molecular and biochemical basis for the biomechanical properties of pelvic floor support tissues. Recent research indicates that reduced collagen content and diminished cross-linking may play critical roles in the onset of POP. Collagen metabolism is closely influenced by matrix metalloproteinases (MMPs) and their inhibitors, known as tissue inhibitors of metalloproteinases (TIMPs). While MMPs primarily promote catabolism (collagen breakdown), TIMPs act as a counterbalance by inhibiting this process. The interplay between MMPs and TIMPs ensures the dynamic equilibrium of the extracellular matrix.

Fibrotic diseases are primarily caused by abnormalities in the connective tissue. The TGF-β/Smad signaling pathway and the Wnt/β-catenin signaling pathway are widely recognized as critical pathways in fibrotic metabolism. Hyperactivation of these pathways has been observed in diseases such as cardiac remodeling, pulmonary fibrosis, renal fibrosis, and systemic sclerosis. Additionally, there are multiple interactions between these two pathways. First, transforming growth factor beta 1 (TGF-β1) can promptly move β-catenin into the cell nucleus in a Smads3-dependent manner. This process regulates the differentiation of mesenchymal stem cells into osteoblasts [6]. Second, TGF-β has the ability to activate the classical Wnt signaling pathway by down-regulating the expression of DKK1, an antagonist of the Wnt pathway. In fibrotic processes regulated by TGF-β, the Wnt/β-catenin pathway is also involved [7]. During tumor metastasis, there is evidence that TGF-β promotes epithelial–mesenchymal transition of tumor cells, activating the Wnt/β-catenin pathway and leading to the expression of related pathway proteins in the tumor cells. In contrast, in diseases like POP, where connective tissue is weakened, several studies suggest that the TGF-β/Smad signaling pathway and the Wnt/β-catenin signaling pathway are inhibited. It is speculated that this inhibition may block collagen synthesis, thereby promoting POP. However, no study has yet reported the correlation between these two pathways and their molecular linkage mechanism in POP. Based on this information, we propose further exploration of the roles of the TGF-β/Smad signaling pathway and Wnt/β-catenin signaling pathway in POP through techniques such as immunohistochemistry, quantitative polymerase chain reaction (qPCR), and western blot analyses. Our goal is to establish a theoretical foundation for future molecular investigations into POP.

## 2 Materials and methods

### 2.1 Research patients

Twenty patients diagnosed with POP of stage III and above, as determined by the POP-Q score were selected as the POP group. Another 20 patients who underwent total hysterectomy for other benign lesions, such as uterine fibroids, benign endometrial lesions, and pre-cancerous cervical lesions, were chosen as the control group. All patients had no history of urinary incontinence, malignant tumors, or connective tissue disease. They had not used estrogen therapy for 3 months before surgery, had no acute or chronic pelvic inflammation, and were confirmed by postoperative pathology to have no endometriosis or adenomyosis. The research was carried out at Laboratory of Otolaryngology-Head and Neck Surgery, the First Hospital of Shanxi Medical University.

Twenty patients in the POP group underwent total hysterectomy + sacroiliac ligament suspension + anterior and posterior vaginal wall repair. Following hysterectomy, a 0.5 × 0.5 × 0.5 cm^3^ piece of vaginal wall tissue was extracted from the midline of the anterior vaginal wall near the fornix during the operation. The tissue was thoroughly rinsed with saline to remove any blood, and then the tissue block was halved using a scalpel. One portion of the tissue block was preserved in 10% neutral formaldehyde solution for histological staining (hematoxylin and Eosin [HE] and Masson staining) and immunohistochemistry experiments. The other portion of the tissue block was frozen at −80°C in a refrigerator for subsequent qPCR and western blot experiments.


**Informed consent:** Informed consent has been obtained from all individuals included in this study.
**Ethical approval:** The research related to human use has been complied with all the relevant national regulations, institutional policies and in accordance with the tenets of the Helsinki Declaration, and has been approved by the Ethics Committee of the First Hospital of Shanxi Medical University (Approval Number: KYLL-2024-132).

### 2.2 Main reagents and instruments

The main reagents and instruments used in the study were Masson trichrome staining kit (Batch No.: G1340-50, Solarbio), TUNEL cell apoptosis kit (Batch No.: MK1015, Boster), TGF-β1 (Batch No.: A16640, Abcolnal), β-catenin (Batch No.: BA0426, Boster), matrix metallopeptidase 2 (MMP2; Batch No.: 10373-2-AP. Wuhan Sanying), TIMP2 (Batch No.: A1558, Abcolnal), collagen type I alpha 1 chain (COL1A1; Batch No.: BA0325, Boster), reverse transcription kit (Batch No.: MF166-Plus-01, Juemei), fluorescence quantification kit (Batch No.: MF787-01, Juemei), enhanced RIPA lysate (Batch No.: AR0102-100, Boster), BCA protein quantification kit (Batch No.: AR0146, Boster), protease inhibitor (Batch No.: AR1178, Boster), 5X protein sampling buffer (Batch No.: AR1112-10, Boster), and electrochemiluminescence (ECL) luminescent liquid (Batch No.: MA0186, Meilun).

### 2.3 Experimental methods

#### 2.3.1 HE and Masson staining

The freshly collected samples of the anterior vaginal wall tissues were immersed in a 10% neutral formaldehyde solution for 48 h. After fixation, 6 μm thick paraffin-embedded sections were prepared. These paraffin sections were dewaxed in xylene and dehydrated in a gradient of ethanol, and HE staining was performed using standard methods. Masson staining was conducted following the instructions provided with the Masson trichrome staining kit. The stained sections were observed using a light microscope.

#### 2.3.2 TUNEL cell apoptosis assay

The sections were conventionally dewaxed and hydrated, then treated with 3% H_2_O_2_ for 10 min, followed by washing with distilled water for 2 min × 3 times. Subsequently, Proteinase K diluted at 1:200 with 0.01 M tris buffered saline (TBS) was applied to the specimens and incubated at 37°C for 10 min, after which the specimens were washed with 0.01 M TBS for 2 min × 3 times. An appropriate amount of labeling buffer was then applied to the tissue to keep it moist. One microliter each of terminal deoxynucleotide transferase and digoxin-d-UTP (digoxin labeled deoxyuridine triphosphate) was mixed with 18 μL of labeling buffer and applied to the sections. The sections were incubated at 37°C in a humid box for 2 h. They were further washed with 0.01 M TBS for 2 min × 3 times. A blocking solution was then applied and incubated at room temperature for 30 min, after which the sections were blotted dry without washing. Biotinylated anti-digoxin antibody, diluted as required, was applied to the tissue and incubated at 37°C for 30 min. The sections were washed with 0.01 M TBS for 2 min × 3 times. The diluted streptavidin-biotin-peroxidase complex was mixed and applied to the sections at 50 μL per slide, and the slides were incubated at 37°C for 30 min. After washing with 0.01 M TBS for 5 min × 4 times, the slides were finally developed with 3,3′-diaminobenzidine (DAB), counterstained with hematoxylin, and observed under a light microscope for staining results. Cells with brown-yellow granules in the nucleus were considered positive cells, indicating apoptotic cells.

#### 2.3.3 Immunohistochemistry

After dewaxing and hydrating the paraffin sections, the antigen was retrieved using citrate buffer. The sections were then treated in the dark with a 3% solution of H_2_O_2_ methanol for 10 min, followed by blocking with 10% goat serum for 20 min. Subsequently, the sections were incubated with the primary antibody and stored overnight in the refrigerator at 4°C. The following day, the primary antibody was removed using phosphate-buffered saline, and the sections were incubated with the secondary antibody in an oven at 37°C for 20 min. The staining process involved using DAB for color development, and the cell nuclei were counterstained with hematoxylin. Finally, the sections were sealed with a neutral mounting medium. The staining was then visualized under a light microscope. The staining results were quantitatively analyzed using Image J software, and the average positive area of the sections was calculated.

Detected proteins: TGF-β1, β-catenin, MMP2, TIMP2, and COLIA1.

#### 2.3.4 Quantitative real-time polymerase chain reaction

In step 1, RNA was extracted using the Trizol method. The concentration and purity of the RNA samples were assessed using a micro-nucleic acid analyzer, and the data were recorded. In step 2, reverse transcription was performed. The reaction system was prepared on ice, after which the reaction mixture was gently mixed and briefly centrifuged to the bottom of the tube, followed by incubation at 42°C for 2 min, and then cooled on ice. The reverse transcription reaction was carried out, and the reaction system was further prepared. The reaction mixture was gently mixed again, briefly centrifuged to collect the liquid at the bottom of the tube, and incubated at 37°C for 15 min; it was then heated at 85°C for 5 s to inactivate the enzyme; finally, it was cooled on ice for the next experiment or stored at −20°C for preservation. Step 3 involved fluorescence quantification using qPCR. The PCR reaction was conducted under the following conditions: initial denaturation at 95°C for 30 s, for one cycle; and 95°C for 3 s, 60°C for 30 s, for a total of 40 cycles. The internal control was human β-actin, and the relative expression of the target gene mRNA in the tissue was calculated using the 2^−ΔΔCT^ method. The sample was centrifuged until all components settled at the bottom of the tube, and then machine detection was carried out. The primer sequences used are shown in Table 1.

**Table 1:** RT**-**qPCR primer sequences

Gene name	Sequence	
	Upstream primer (5′–3′)	Downstream primer (5′–3′)
TGF-β1	TATTGAGCACCTTGGGCACT	ACCTCTCTGGGCTTGTTTCC
β-catenin	CGGGCAAGCCAGATGTTTAT	CGCCACCTTCTTTGTTCAGTTT
MMP2	AGTTTCCATTCCGCTTCCAG	CGGTCGTAGTCCTCAGTGGT
TIMP2	TCTGGAAACGACATTTATGG	GTTGGAGGCCTGCTTATGGG
COL1A1	CAAGACGAAGACATCCCACCAATC	ACAGATCACGTCATCGCACAACA
β-actin	CCTGGCACCCAGCACAAT	GGGCCGGACTCGTCATAC

#### 2.3.5 Western blot

A solution containing enhanced RIPA lysate and protease inhibitor was prepared and pre-cooled at a volume ratio of 100:1. Fifty milligrams of tissue was weighed and placed in the pre-cooled lysate. The tissue was then ground in a grinder and lysed on ice for 30 min until the tissue mass morphology disappeared into a paste. Subsequently, the lysate was centrifuged at 10,000*g* for 10 min in a pre-cooled centrifuge at 4°C, and the supernatant was collected as the protein. Protein quantification was performed using the BCA protein quantification kit. The protein samples were then mixed with 5× protein sampling buffer at a volume ratio of 4:1 and the mixture was boiled at 100°C for 10 min. Subsequently, the samples were stored in the refrigerator at −20°C for future use. Before the experiment, the samples were thawed, uploaded, electrophoresed, and transferred to membranes. The membranes were then placed in 10% skimmed milk powder at room temperature in a closed shaker for 2 h. Following this, the primary antibody was incubated at 4°C overnight. The following day, the membranes were washed three times with TBST solution. The secondary antibody was then incubated at room temperature for 2 h, followed by another three washes with TBST solution. The proteins were visualized using the ECL luminescent solution, and the gray values of the images were analyzed with Image J software to create a bar graph.

The concentrations of primary antibodies used were as follows: TGF-β1 (1:1,000), β-catenin (1:1,000), MMP2 (1:1,000), TIMP2 (1:1,000), COL1A1 (1:1,000), and β-actin (1:8,000) and secondary antibody (1:15,000).

#### 2.3.6 Statistical analysis

Statistical Package for the Social Sciences (SPSS) 27 software was used for data statistics, and GraphPad Prism 9.5.0 software was used for graphing. The measurements were expressed as mean ± standard deviation. Comparisons between groups were analyzed by *t*-test if they followed a normal distribution, and Mann–Whitney analysis was used if they did not follow a normal distribution. A significance level of *p* < 0.05 indicated statistically significant differences. Correlation tests were conducted based on the distribution of the data. If the data followed a normal distribution, Pearson’s correlation analysis was used. If the data did not follow a normal distribution, Spearman’s correlation analysis was employed. Significant differences in the graphs are indicated by *p* < 0.05 (*), *p* < 0.01 (**), *p* < 0.001 (***), and *p* < 0.0001 (****).

## 3 Results

### 3.1 General data

The general information of the 40 patients included in the study is summarized in Table 2. The age, number of pregnancies and births, and body mass index of the two groups were calculated. The differences between the respective comparisons were not statistically significant (*p* > 0.05).

**Table 2:** Comparison of general information between the two groups

Group	*n*	Age (years)	Number of pregnancies	Number of births	BMI (kg/m^2^)
POP group	20	53.45 ± 6.67	2.85 ± 0.93	1.95 ± 1.00	23.75 ± 2.42
Control group	20	50.75 ± 6.80	2.50 ± 1.10	1.55 ± 0.69	23.23 ± 2.42
*t*		1.27	1.09	1.48	0.77
*P*		0.213	0.285	0.148	0.448

### 3.2 HE and Masson staining

**Figure 1:**
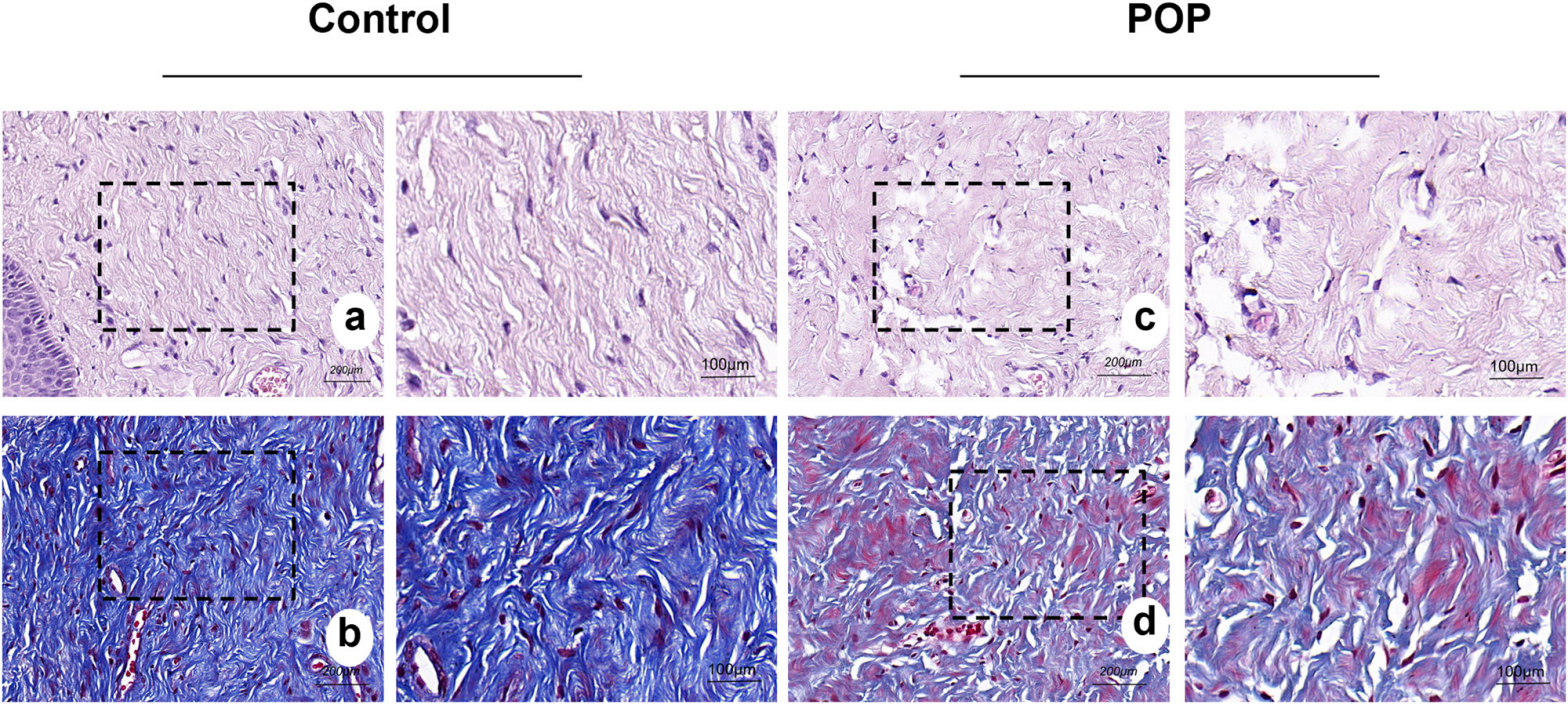
HE staining and Masson staining of the tissue of the anterior vaginal wall: (a) and (b) anterior vaginal wall of the control group; (c) and (d) anterior vaginal wall of the POP group. Collagen fibers were pink in HE staining and blue in Masson staining. (a)–(d) magnification 50×.

The staining results (Figure 1) revealed that the collagen fibers in the tissue of the anterior vaginal wall of the control group were arranged in an orderly manner and tightly aggregated into bundles. In contrast, the collagen fibers in the POP group appeared disorganized, loosely structured, and exhibited more breaks. Tissue components on the slide are indicated in Figure S1.

### 3.3 TUNEL cell apoptosis assay

**Figure 2:**
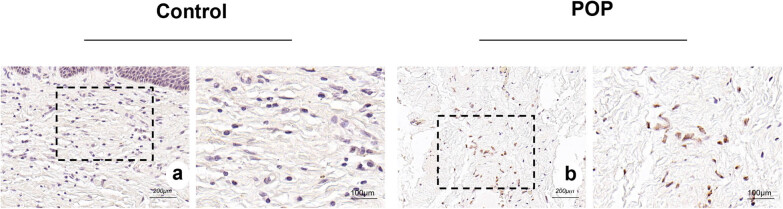
(a) and (b) TUNEL cell apoptosis staining of the tissue of the anterior vaginal wall in the control and POP groups, respectively. The brownish yellow granules are nuclei of the apoptosis cells. (a) and (b) magnification 50×.

The nuclei of apoptosis cells were brownish-yellow in color, and the staining results showed that there were significantly more apoptosis cells in the anterior vaginal wall tissue of the POP group than in that of the control group (Figure 2).

### 3.4 Immunohistochemistry

**Figure 3:**
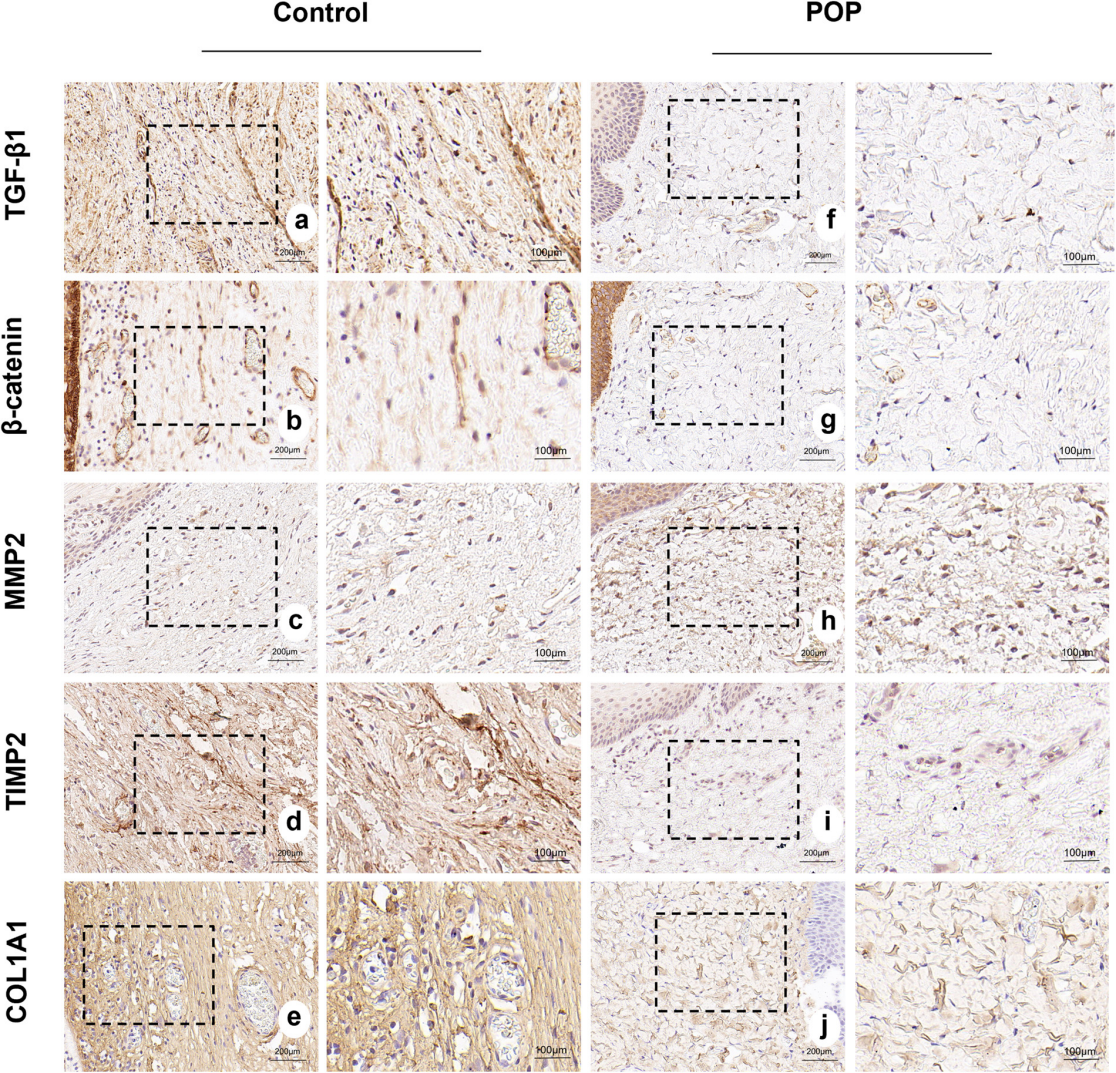
(a)–(e) Expression of TGF-β1, β-catenin, MMP2, TIMP2, and COL1A1 in the anterior vaginal wall tissues of patients in the control group. (f)–(j) Expression of TGF-β1, β-catenin, MMP2, TIMP2, and COL1A1 in the anterior vaginal wall tissues of patients in the POP group, respectively. (a)–(j) magnification 50×.

**Figure 4:**
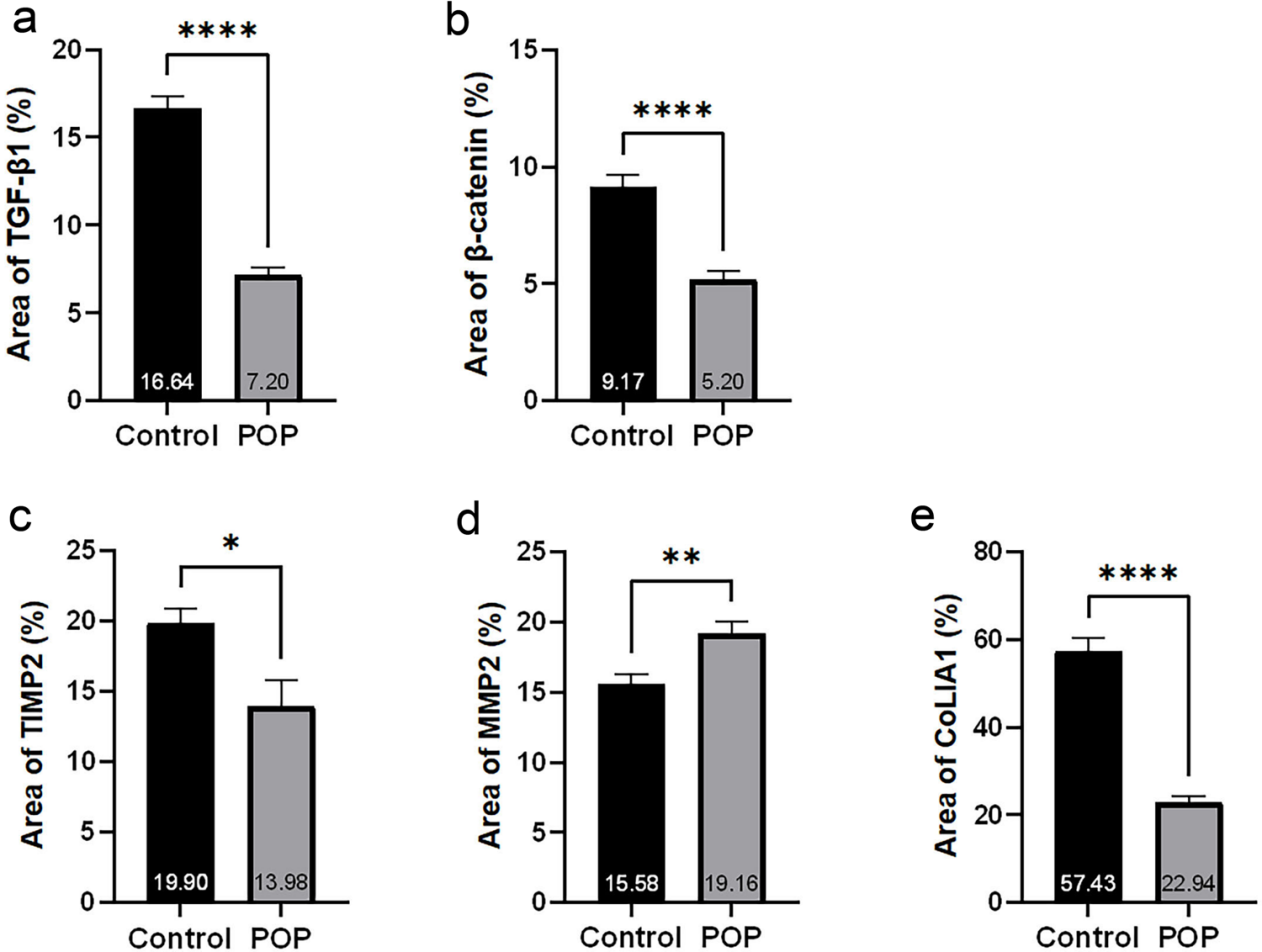
(a)–(e) Statistical results of immunohistochemical staining of TGF-β1, β-catenin, MMP2, TIMP2, and COL1A1, respectively.

The results showed that collagen I was mainly expressed in the intercellular matrix, and the expression of collagen I in the POP group was significantly lower than that in the control group. MMP2 and TIMP2 were primarily expressed in the interstitium. We observed a significantly higher expression of MMP2 in the POP group compared to the control group, whereas TIMP2 showed a significantly lower expression in the POP group than in the control group. β-catenin was expressed in both the nucleus and the cytoplasm, with predominant expression in the nucleus. The expression in the POP group was significantly lower than that in the control group. TGF-β1 was primarily expressed in the cytoplasm, and to a lesser extent in the nucleus. Its expression in the tissue of the anterior vaginal wall of the POP group was significantly lower than that of the control group. All the aforementioned differences were statistically significant (Figures 3 and 4).

### 3.5 Quantitative real-time polymerase chain reaction

**Figure 5:**
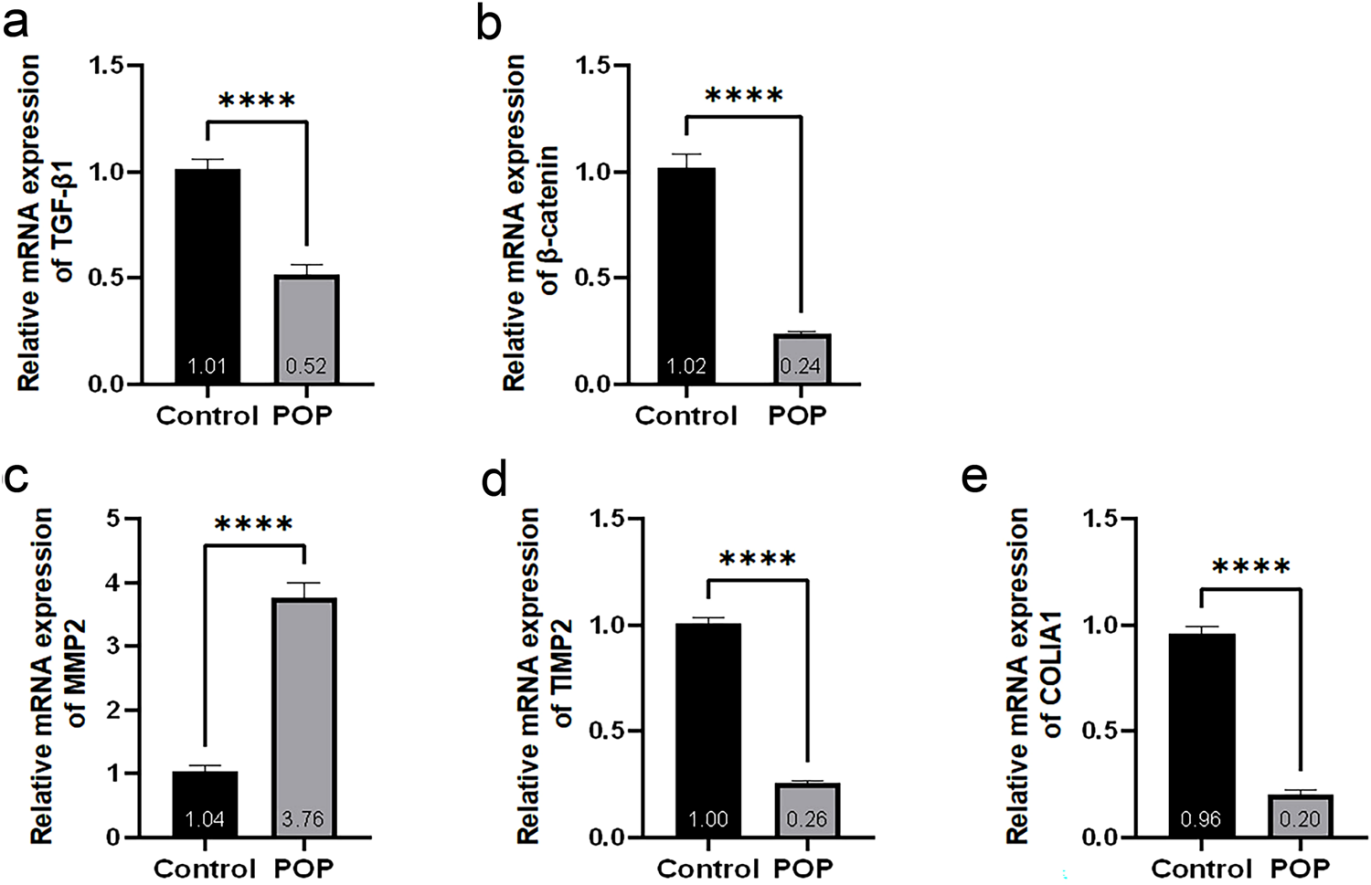
(a)–(e) Statistical results of TGF-β1, β-catenin, MMP2, TIMP2, and COL1A1 mRNA expression levels in the anterior vaginal wall tissues of control and POP groups.

The results revealed that the mRNA expression levels of TGF-β1, β-catenin, TIMP2, and COL1A1 in the POP group were significantly lower than those in the control group. In contrast, the expression of MMP2 was significantly higher in the POP group compared to the control group, and these differences were statistically significant (Figure 5).

### 3.6 Western blot

**Figure 6:**
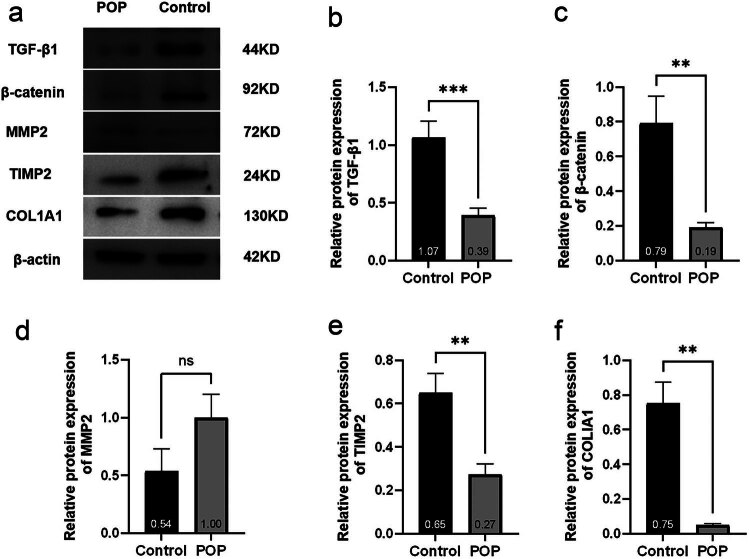
(a) Strip graph of the western blot and (b)–(f) statistical analysis results of western blot.

The results indicated that the protein expression levels of TGF-β1, β-catenin, TIMP2, and COL1A1 in the tissue of the anterior vaginal wall of the POP group were significantly lower than those of the control group. The differences were statistically significant, with levels of difference at ***, **, **, and **, respectively. Although the expression of MMP2 was higher than that of the control group, the difference was not statistically significant (*p* > 0.05, Figure 6).

### 3.7 Correlation analysis

**Figure 7:**
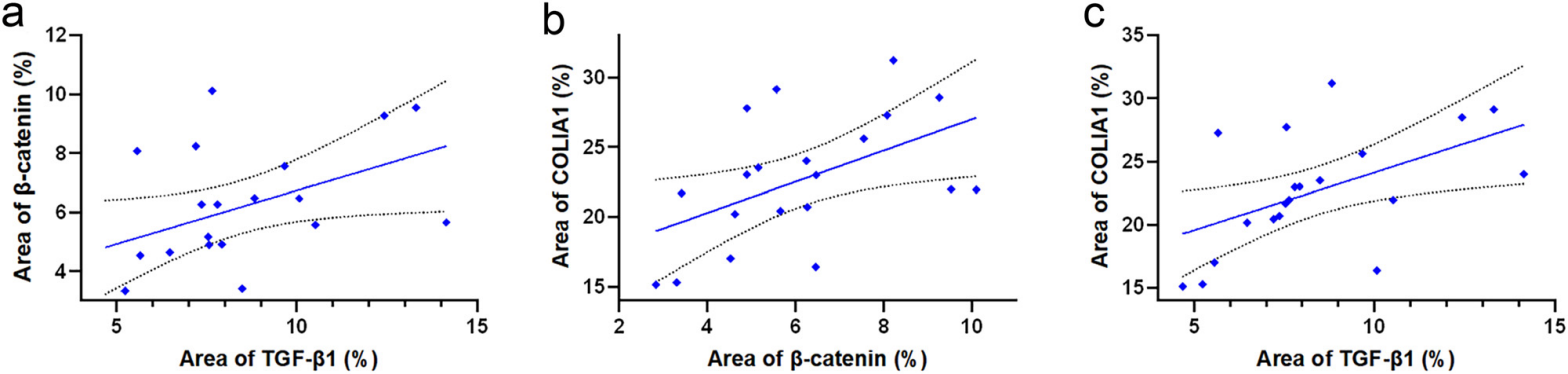
(a) Correlation test between the immunohistochemical staining results of TGF-β1 and β-catenin, *r* = 0.46, *p* < 0.05. (b) Correlation test between the immunohistochemical staining results of COL1A1 and β-catenin, *r* = 0.50, *p* < 0.05. (c) Correlation test between COL1A1 and TGF-β1 and immunohistochemical staining results, *r* = 0.62, *p* < 0.05.

Examine the correlation of protein expression levels of TGF-β1, β-catenin, and COL1A1 in patients of the POP group. Considering the small sample size of the study, Spearman correlation test was used, revealing a significant positive correlation between TGF-β1 and β-catenin, β-catenin and COL1A1, as well as TGF-β1 and COL1A1 in the tissues of the anterior wall of the vagina (Figure 7).

## 4 Discussion

In this study, we found that collagen fibers in the tissue of the anterior vaginal wall of patients with POP were disorganized in orientation, diminished in quantity, and fractures were notably prevalent. Meanwhile, TUNEL cell apoptosis staining revealed a significantly higher number of apoptotic cells in the vaginal wall of the POP group compared to the control group. The results of immunohistochemistry, qPCR, and western blot all showed that patients in the POP group had significantly lower levels of type I collagen than those in non-POP patients. These results suggest that the decreased amount and structural abnormality of collagen may be related to the onset of POP. POP is commonly recognized as a condition characterized by diminished pelvic floor tissue support resulting from aging, childbirth, and connective tissue abnormalities. Collagen, an important component of connective tissue, plays a key role in supporting the pelvic floor. Zhu et al. [8] detected the expression of type I collagen and type III collagen in the uterine sacral ligament of 35 POP patients and 20 control patients. The experimental results showed that the expression levels of type I collagen and type III collagen in the sacral ligament tissues of the POP group were decreased, and the expression levels of the genes Bax and Bad related to apoptosis were up-regulated. It is suggested that POP is related to the remodeling of extracellular matrix in the uterine and sacral ligaments. The main pathological features are the decrease of collagen synthesis, the increase of collagen decomposition, and the increase of apoptosis level. This result is consistent with our experimental results, so it can be suggested that the occurrence of POP may be related to the imbalance of collagen metabolism in the pelvic floor supporting tissue. In addition, enzymes related to collagen metabolism, such as MMPs and TIMPs, play an important role in maintaining the dynamic remodeling of the extracellular matrix.

MMP2 is a crucial enzyme in the metabolism of the extracellular matrix and is closely associated with the remodeling of the extracellular matrix in both physiological and pathological conditions. MMP2 is also known as gelatinase A. The main hydrolysis products are collagen type IV and collagen type V, among others. The degradation function of MMP2 is regulated by its specific antagonist TIMP2. TIMP2 inhibits the degradation of extracellular matrix proteins by MMP2. It achieves this primarily by binding non-covalently to the activated MMP2 molecules in a 1:1 ratio, effectively inactivating them. Previous studies have shown that MMP2 expression is higher and TIMP2 expression is lower in sacral ligament tissues of patients with POP, the results indicated that the degradation of collagen in sacral ligament tissues of POP patients was higher than that of control group. In this study, the expression levels of MMP2 and TIMP2 in the anterior vaginal wall tissues were detected, and the same results were obtained. However, a recent study [9] found that the expression of MMP2 in the vaginal wall tissue of the POP group was significantly up-regulated, which was consistent with our results. However, although the expression of TIMP1 was reduced, there was no statistical difference between it and the control group, which we speculated might be caused by individual differences or insufficient sample size. It is hoped that this problem can be proved by establishing uniform sample acquisition standards and expanding sample size in the future. Combined with the fact that the expression of type I collagen is reduced and disorganized in its orientation in the vaginal wall of patients in the POP group, it is reasonable to hypothesize that the increased catabolism and decreased synthesis of collagen are important factors contributing to the development of POP.

TGF-β has been widely reported as an important regulator of fibrotic metabolism in fibrotic and degenerative diseases [10,11]. TGF-β is a multifunctional protein polypeptide. It exists in four isoforms: TGF-β1, TGF-β2, TGF-β3, and TGF-β1β2. These isoforms not only promote a large amount of collagen synthesis and secretion from fibroblasts, but also enhance collagen synthesis and reduce collagen degradation by up-regulating TIMPs and inhibiting the activity of MMPs. Research indicates that in early-passaged human gingival fibroblasts, TGF-β1 plays a role in regulating the activity of MMP2. Specifically, it decreases the mRNA level of MMP2 and increases the mRNA level of TIMP [12]. Another study found that the expression level of TGF-β1 in sacral ligament tissues of patients with POP was significantly lower compared to that in non-POP patients. Additionally, the expression level of TGF-β1 mRNA was partially negatively correlated with the severity of POP [13]. These findings align with our own experimental results. Our investigations, including immunohistochemistry, qPCR, and western blot analyses, consistently demonstrated that the expression of TGF-β1 in the tissue of the anterior vaginal wall of patients in the POP group was significantly lower than that in the control group. Based on these collective findings, we hypothesize that TGF-β1 may play a role in the development of POP by regulating the metabolism of the extracellular matrix. However, a recent study on the correlation between vaginal microecological changes and pelvic floor collagen metabolism in POP patients found [13] that mRNA expressions of Decorin, TGF-β1, and matrix metalloproteinase-3 were increased in sacrum ligament tissues of POP patients. The mRNA expression of type I and III collagen decreased. According to the different research results of TGF-β1, we speculated that the expression and function of TGF-β1 may be affected by factors such as research method, material location, study population, sample size, and genetic background.

The Wnt/β-catenin signaling pathway plays a significant role in a variety of tissue repair and fibrotic diseases. It is an important pathway in collagen synthesis [14]. β-catenin, a key member of this classical Wnt pathway, plays a crucial role in regulating cell differentiation, proliferation, and apoptosis. In a study by Gong et al. [15] silencing β-catenin in vaginal wall fibroblasts of non-POP patients resulted in a significant decrease in cell proliferation rate and a reduction in type I collagen expression. Conversely, activating the Wnt/β-catenin signaling pathway with lithium chloride yielded results opposite to the previous findings. Immunofluorescence results indicated an increase in the nuclear translocation of β-catenin. As a transcriptional co-activator, β-catenin initiates the expression of target genes downstream of the classical Wnt pathway by binding to transcription factors TCF/LEFs [16]. These factors form a transcriptional complex that can bind to the promoter sequence of the COL3a1 gene, which is directly involved in regulating extracellular matrix-related genes [17]. Our results demonstrated that the levels of β-catenin protein expression and gene transcription in the anterior vaginal wall tissues of patients with POP were significantly lower than those of non-POP patients. This suggests that the reduced expression of β-catenin may be another crucial factor contributing to the development of POP.

The TGF-β/Smad pathway and the Wnt/β-catenin pathway work together in various fibrotic diseases [18,19], including cardiac remodeling, pulmonary fibrosis, and renal fibrosis. They play a role in regulating cell fate and are involved in collagen metabolism. Increasing evidence suggests that TGF-β-induced fibrosis is closely related to the Wnt signaling pathway, and they are jointly involved not only in the regulation of fibrotic diseases but also in the regulation of tumor invasion and metastasis [20,21]. However, to the best of our knowledge, the interaction between the two has not been thoroughly studied in patients with POP. For this reason, we analyzed the correlation between the key factors of the two signaling pathways, TGF-β1 and β-catenin, as well as the protein expression of each with COL1A1 in the tissue of the anterior vaginal wall of patients with POP. The immunohistochemical staining results of the anterior vaginal wall of the patients were subjected to Spearman correlation test. The analysis revealed a significant positive correlation among the protein expression levels of TGF-β1, β-catenin, and COL1A1. This finding supports our hypothesis and establishes a solid theoretical foundation for our upcoming *in vitro* cellular experiments.

## 5 Conclusion

In this study, we demonstrated that the level of cellular necrosis in the vaginal wall tissues of patients with POP was higher than that of the control group. Additionally, we found that the expression level of type I collagen was significantly down-regulated. Moreover, the expression of MMP2, which promotes collagen degradation, had increased, while the expression of TIMP2, which inhibits collagen degradation, had decreased. In addition, we demonstrated that the expression of TGF-β1 and β-catenin in the vaginal wall tissue of patients with POP was significantly lower than that of the control patients, and the protein expression levels of the two showed a strong correlation. These data suggest that POP may occur due to the inhibition of the TGF-β/Smad signaling pathway and the Wnt/β-catenin signaling pathway. This inhibition leads to a decrease in collagenous components and an increase in cellular apoptosis in connective tissues. Additionally, the two pathways are interconnected and crosstalk with each other. Abnormalities in TGF and Wnt signaling pathways are commonly observed in various connective tissue diseases. By investigating the interaction between TGF-β and Wnt signaling pathways, we aim to develop targeted therapeutic drugs that effectively manage the progression of POP. This approach could potentially benefit women with POP, helping them regain confidence and improve their quality of life.

## 6 Limitations

It is essential to acknowledge the limitations of this study. First, the sample size is relatively small; hence, we plan to conduct multi-center clinical studies in the future to gather more robust data. Second, this study is retrospective, which makes it challenging to definitively establish whether the decrease in TGF-β1 and β-catenin expression directly causes POP or if POP itself leads to reduced expression of these factors based solely on this experiment. To address this, we intend to further investigate the causal relationship between these pathways through additional *in vitro* experiments.

## Supplementary Material

Supplementary Figure
