# Supplementary figures and images for: Association between TGF-β1 and β-catenin expression in the vaginal wall of patients with pelvic organ prolapse
Source: Open Life Sci. 2025 Mar 21;20(1):20221058. doi: 10.1515/biol-2022-1058 (PMC11931662; doi:10.1515/biol-2022-1058)

# Supplementary material

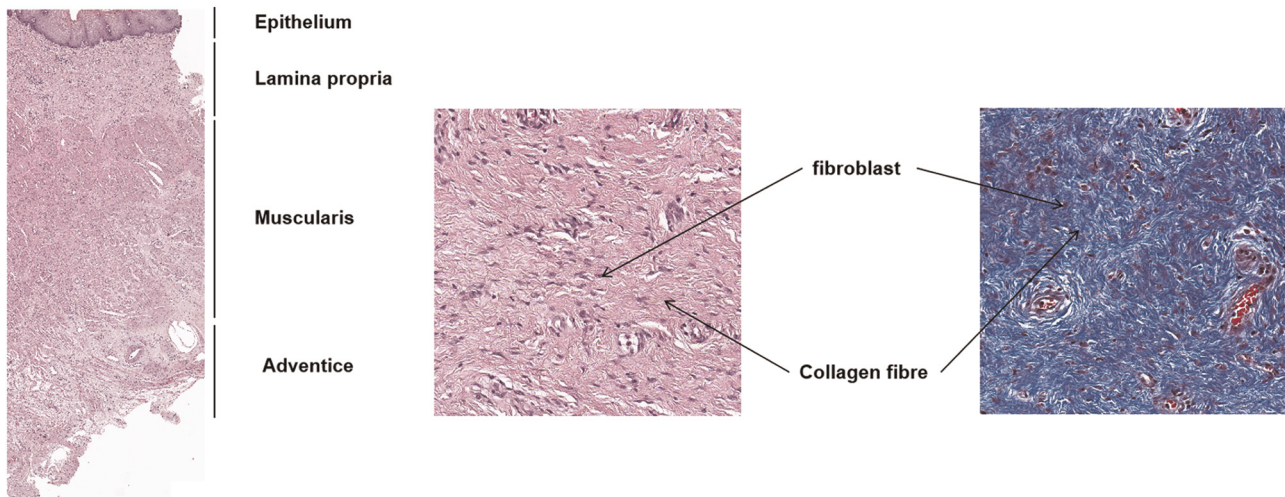

**Figure S1:** Tissue components on the slide were indicated.

Supplement: Supplementary Figure [file biol-2022-1058-sm.pdf]
